# Exploring porphyrins induced carbon nanocone TM-PICNC (TM = Sc^2+^, Ti^2+^, V^2+^, Cr^2+^, Fe^2+,^ Co^2+^, Ni^2+^, Cu^2+^, and Zn^2+^) as a highly sensitive sensor for CO_2_ gas detection in presence O_2_ and H_2_O molecules: a computational study

**DOI:** 10.3389/fchem.2023.1305362

**Published:** 2023-11-28

**Authors:** Guizhou Wu, Sattar Arshadi, Omid Pouralimardan, Vahideh Abbasi, Esmail Vessally

**Affiliations:** ^1^ School of Measurement and Communication Engineering, Harbin University of Science and Technology, Harbin, China; ^2^ Heilongjiang Institute of Construction Technology, Harbin, China; ^3^ Department of Chemical Engineering, University of Science and Technology of Mazandaran, Behshahr, Iran; ^4^ Department of Chemistry, Payame Noor University, Tehran, Iran; ^5^ Department of Chemistry, University of Zanjan, Zanjan, Iran

**Keywords:** CO_2_, sensor, nanocone, porphyrin, DFT

## Abstract

This study investigated the adsorption of CO_2_ molecules on transition metal ions (TM) porphyrins induced carbon nanocone (TM-PICNC) (TM = Sc^2+^, Ti^2+^, V^2+^, Cr^2+^, Fe^2+,^ Co^2+^, Ni^2+^, Cu^2+^, and Zn^2+^) using density functional theory (DFT) to determine the stabilities, energetic, structural, and electronic properties. The results showed that the CO_2_ molecule is adsorbed on TM-PICNC with adsorption energies ranging from 0.03 to −12.12 kcal/mol. The weak interactions of CO_2_ gas with Cr, Ni, Cu, and Zn-PICNC were observed, while strong adsorption was found on Sc, Ti, and V-PICNC. The Ti, V, and Cr-PCNC structures were shown to have a suitable energy gap (E_g_) for sensing ability because of the effective and physical interaction between these structures and CO_2_ gas, leading to a short recovery time. DFT calculations also revealed that V-PCNC had a high %ΔE_g_ (about %56.79) and hence high sensitivity to CO_2_ gas, making it a promising candidate for having good sensing ability to CO_2_ gas in presence of O_2_ and H_2_O gas.

## Introduction

The issue of global warming and glacier melting has been a major environmental concern, largely attributed to the rising concentration of carbon dioxide (CO_2_) in the atmosphere. To address this problem, researchers have undertaken various efforts to detect, transform, or store CO_2_ (Waghuley, 2011; Fan et al., 2013; Van Hieu et al., 2013; Kannan et al., 2014; Zhu et al., 2017; Wang et al., 2019). Capturing and storing CO_2_ is a significant step towards mitigating the effects of climate change and promoting the utilization of CO_2_. This process has the potential to make a significant contribution to improving the global climate by reducing the amount of CO_2_ released into the atmosphere. Additionally, with the development of new technologies, CO_2_ can be used as a valuable resource for various industrial applications, further emphasizing the importance of CO_2_ capture and storage. The use of solid sorbents to capture CO_2_ is a highly promising technique for reducing CO_2_ levels (Yang et al., 2008). This method has numerous advantages, including its eco-friendliness, cost-effectiveness, noncorrosive nature, high gas capacity, and low regeneration energy requirements (Chen et al., 2009; Choi et al., 2009). Hence, there is a pressing need to develop new materials that possess a high capacity for capturing CO2.

Porphyrins are a type of aromatic macrocycle organic structure that serves a crucial role in biological functions like respiration (Poulos, 2014), electron transport (Yoshikawa and Shimada, 2015) and photosynthesis (Scheer, 2006). These structures are comprised of four functionalized pyrroles connected by methine bridges (=CH-). Porphyrins and other compounds derived from them have a range of applications in medicine, the energy sector, and the chemical industry (Kingsbury and Senge, 2021). Due to their strong light absorption capabilities, porphyrins have been investigated for use in photodynamic therapy (PDT), a non-invasive cancer treatment (Králová et al., 2010; Xue et al., 2019). Haver and Anderson have examined different strategies to synthesize fully π-conjugated monodisperse porphyrin nanotubes with either butadiyne (C4) or acetylene (C2) links between the conjoined 6-porphyrin nanorings (Haver and Anderson, 2019). The porphyrin nanocone (nanocone cap) and porphyrin nanotube have been synthesized by A. Uka in 2020. The porphyrin nanocone has been synthesized by a reaction between isoindole and triphenylbenzaldehyde to yield benzoporphyrin (nanocone precursor). Then, the precursor molecule has been converted to the respective seeds by surface-assisted cyclodehydrogenation on Pt(111) surface and subsequently grown to the porphyrin nanocone (Uka, 2020). Additionally, certain porphyrin-based compounds have been studied for their potential as anticancer and antioxidant materials (Bajju et al., 2019). Metalloporphyrins are porphyrin cycles that have had metal ions inserted into them. These compounds have been extensively studied for use as (photo) catalysts in organic synthesis, oxidation of organic compounds, and photocatalytic water splitting (Huang and Groves, 2017; Zhang et al., 2017). Additionally, the aggregation of metalloporphyrins onto TiO2 has been evaluated for use in dye-sensitized solar cells (Mojiri-Foroushani et al., 2013; Mendizabal et al., 2017). The application of metalloporphyrins as chemical sensors has garnered significant attention (D'Amico et al., 2000; Rakow and Suslick, 2000; Salleh and Yahaya, 2002; Tao et al., 2006; Amao and Okura, 2009). Tao et al. (2006) investigated the potential of metalloporphyrins as sensors for rapidly detecting trace amounts of TNT vapors. Lee et al. (2021) on the other hand, utilized metalloporphyrin-functionalized reduced graphene oxide for analyzing human breath and detecting volatile organic compounds. Carbon nanomaterials have garnered significant interest among researchers due to their exceptional properties and remarkably high surface area. Consequently, they have been extensively studied for their diverse applications, including gas adsorption, detection, catalysis, and more (Llobet, 2013; Babu et al., 2017; Bashiri et al., 2017; Babu et al., 2018; Ganazzoli and Raffaini, 2019; Gusain et al., 2020; Vessally et al., 2021; Jouypazadeh et al., 2023; Söğütlü et al., 2023). Tripkovic et al. (2013) synthesized metalloporphyrin-like graphene and utilized it for the electrochemical reduction of CO_2_ and CO. Khalif et al. (2023) explored the potential of the transition metals (TM(II) = Sc^2+^, Ti^2+^, V^2+^, Cr^2+^, Mn^2+^, Fe^2+,^ Co^2+^, Ni^2+^, Cu^2+^ and Zn^2+^) porphyrins induced carbon nanocone (TM(II) PICNC) for adsorbing and detecting O_2_ gas. The aim of this study was to investigate the stabilities and electronic properties of carbon nanocones induced by first-row transition metal ions (TM = Sc^2+^, Ti^2+^, V^2+^, Cr^2+^, Fe^2+,^ Co^2+^, Ni^2+^, Cu^2+^ and Zn^2+^)-porphyrin (TM-PICNC), as well as their interactions with CO_2_ on both the interior and exterior surfaces. The study utilized density functional theory (DFT) calculations to explore the potential of TM-PICNCs as nanosensors.

Over the years, different classes of carbon capture materials have been identified. For example, Songolzadeh et al. (2012) categorized CO_2_ adsorbents into two classes: physical and chemical adsorbents. Comparatively, physical adsorbents offer significant benefits for energy efficiency over chemical and physical absorption routes. The adsorption process involves either physisorption (van der Waals) or chemisorption (covalent bonding) interactions between gas molecules and the material’s surface. When dealing with physical adsorbents, balancing the solid affinity for removing undesired components from a gas mixture with the energy consumption required for regeneration is crucial. Additionally, selectivity is another relevant factor that affects adsorptive gas separation, in addition to the adsorption capacity. Various physical adsorbents have been investigated for CO2 capture, including metal oxides, hydrotalcite-like compounds, microporous and mesoporous materials such as activated carbon and carbon molecular sieves, zeolites, and chemically modified mesoporous materials [as detailed in references (Choi et al., 2009; Zhao et al., 2010a; Zhao et al., 2010b; Akhtar et al., 2012)]. Physical adsorbents, also known as physisorbents, are minimally affected during adsorption. The use of solid sorbents to capture CO_2_ is a highly promising technique for reducing CO_2_ levels. This method has numerous advantages, including its eco-friendliness, cost-effectiveness, noncorrosive nature, high gas capacity, and low regeneration energy requirements (Wang et al., 2019).

Activated carbon, charcoal, coal, and carbon nanomaterials are some of the carbon-based materials that have been studied for high-pressure CO_2_ capture applications (Mazumder et al., 2006; Choi et al., 2009; Mahyoub et al., 2022). These materials offer several advantages, such as low cost, insensitivity to moisture, and the possibility of producing or synthesizing them from various naturally existing or spent carbon-based materials (Plaza et al., 2012). Due to their significantly high surface area, they have a greater adsorption capacity at high pressures, making them suitable for a range of high-pressure gas separation applications.

In an experimental investigation, the study involved the preparation of electrodes modified with iron porphyrin and carbon nanotubes (FeP-CNTs) for the purpose of CO_2_ electro-reduction. The adsorption of iron porphyrin onto the multi-walled carbon nanotubes was examined using scanning electron microscopy and ultraviolet and visible spectroscopy. The electrochemical properties of the modified electrodes for CO_2_ reduction were evaluated using cyclic voltammetry and CO_2_ electrolysis. The FeP-CNT electrodes demonstrated a less negative cathode potential and a higher reaction rate compared to the electrodes modified solely with iron porphyrin or carbon nanotubes. The research sheds light on the mechanism of synergistic catalysis between CNTs and metallo-porphyrin. Based on the findings, the iron porphyrin-CNT modified electrodes exhibit promising potential for efficient CO_2_ electro-reduction (Zhao et al., 2013).

A comparative study of electrocatalytic CO_2_ reduction was conducted using cobalt meso-tetraphenylporphyrin (CoTPP) as a model molecular catalyst under both homogeneous and heterogeneous conditions. The study found that when CoTPP was immobilized onto carbon nanotubes, its electro-catalytic abilities were significantly enhanced, resulting in selective reduction of CO_2_ to CO (>90%) at a low over potential in an aqueous medium. This effect was attributed to the specific environment created by the aqueous medium at the catalytic site of the immobilized catalyst, which facilitated the adsorption and further reaction of CO_2_. The research highlights the importance of evaluating an immobilized molecular catalyst beyond homogeneous measurements alone (Hu et al., 2017).

Another experimental research study documented the creation of a novel three-dimensional (3D) polypyrrole nanocone membrane, which boasted an exceptionally large surface area of 949.5 m^2^/g - surpassing all previously reported data (El-Said et al., 2022). The researchers employed a straightforward, *in situ*, template-free electrochemical technique to fabricate the nanocone membrane, without requiring a highly alkaline environment. The study also analyzed the chemical composition and morphology of the resulting membrane. The membrane demonstrated high affinity for CO_2_ and exhibited hydrophobic properties, preventing moisture adsorption. These results demonstrated that the synthesized 3D nanocone has a CO_2_ gas storage capacity of approximately 68 mg/g and can be regenerated without the need for heat application.

In another study, the effects of N-doping on the CO_2_ adsorption properties of carbon materials were investigated for the first time using carbon nanotubes (CNTs) (Babu et al., 2017). CNTs are excellent model structures for studying gas adsorption, as they possess a well-defined, reproducible mesoporous pore structure and a chemically uniform surface, thereby avoiding the confounding effects of micropores that are commonly found in carbon-based adsorbents. The presence of nitrogen functionalities was found to have a positive impact on CO_2_ adsorption over a wide pressure range (0–36 bar). The nature of the interaction was determined by calculating the isosteric heat of adsorption. Furthermore, the importance of determining the oxygen functional groups in the adsorbent was highlighted by comparing the adsorption characteristics of as-prepared and N-doped CNTs with oxygen-functionalized CNTs. All these motivated us to explore theoretically the formation of TM cations porphyrin induced carbon nanocone (TM-PICNC) and the adsorption of CO_2_ molecule over these functionalized carbon nanocone.

## Computational details

The selected model system consists of nanocones with a cone angle of 83.6° (disclination angle of 120) and a length of 8.46 Å, composed of 81 carbon atoms and 20 hydrogen atoms. Unlike the nanocone with 120 disclination angle, it is not possible to insert the porphyrin ring in nanocones with other angles (60, 240, and 300). All calculations were conducted at the DFT level using hybrid density functional (B3LYP) (Becke, 1993) and 6-31G(d) basis set(Ochterski et al., 1996) in the gas phase with the GAMESS software (Schmidt et al., 1993). Previous studies (Baei, 2012; Hizhnyi et al., 2017; Soleimani-Amiri, 2017) utilized the B3LYP functional for theoretical investigations, while a similar work (Baei et al., 2012) employed the 6–31 g(d) basis set. After the full optimization and obtained all considered configurations, single point calculations B3LYP/CC-PVTZ level of theory (Dunning Jr, 1989; Kendall et al., 1992) are performed for favorable configurations (CNC, PICNC and V-PICN configurations) to obtain more accurate results. To create a defect in the carbon nanocone, a PI ring with 4 nitrogen atoms and 20 carbon atoms was inserted at the tip of the cone, similar to the structure of PI. The Gaussview software was used to replace the PI structure with the defect in the carbon nanocones (PICNC). The optimization calculations were then performed on the complete Nanocone and the PICNC structure. DOS plots were generated using the GaussSum program (O’boyle et al., 2008).

The adsorption energies of the molecules on the surface were obtained using the following equation:

$$E_{\text{ads}}=E_{\left(\text{Gas}/\text{TM}-\text{PICNC}\right)}-\left[E_{\left(\text{TM}-\text{PICNC}/\text{Gas}\;\text{ghost}\right)}+E_{\left(\text{Gas}/\text{TM}-\text{PICNC}\;\text{ghost}\right)}\right]+E_{\text{BSSE}}$$ (1)

In the provided equation, the term E_ads_ refers to the adsorption energy of a CO_2_ molecule onto a TM-PICNC molecule. The term E_(Gas/TM-PICNC)_ represents the total energy of interaction between the CO_2_ gas and the TM-PICNC molecule, while E_(TM-PICNC/Gas ghost)_ refers to the energy of the TM-PICNC molecule. The term E_(Gas/TM-PICNCF ghost)_ represents the energy of the CO_2_ molecule. Counterpoise correction is a technique commonly employed to correct for the basis set superposition error (Turi and Dannenberg, 1993). This method involves calculating the energy of the complex first and then performing separate calculations for the individual molecules using the same basis set functions as in the complex. Although counterpoise correction is not theoretically necessary for large basis sets, it has been observed to produce significant improvements in accuracy in practice, especially for such sets. The Natural Bond Orbitals (NBO) charge analysis was performed at the same level of theory. In this study, the HOMO-LUMO energy gap (E_g_) is defined as:

$$E_{g}=E_{\text{LUMO}}-E_{\text{HOMO}}$$ (2)

In this context, the energy of the HOMO and LUMO orbitals are denoted as E_HOMO_ and E_LUMO_, respectively. The Fermi level (E_F_) is conventionally assumed to be located approximately at the middle of the energy gap (E_g_) of the molecule at 0 K.

Density Functional Theory (DFT) Calculations were performed to examine the potential for competing reactions involving residual gases such as H_2_O and O_2_. The results of the DFT calculations indicated that the interactions between these gases and the studied nanostructures did not play a competitive role in these structures.

In this work, we theoretically studied in a non-periodic approach. Unlike the periodic approach, which calculates the phonon spectrum for solid or liquid systems by considering vibrational modes at each k-point, frequency calculations are performed to determine the vibrational modes in the non-periodic approach. Our frequency calculations revealed that all structures in our system are located in local minima of the potential energy surface, as indicated by the absence of vibrational modes with imaginary values. Therefore, we conclude that the structures are stable.

One of the critical tasks in the electronics industry is to regulate the work function of materials to enhance device performance. Controlling the work function of nanomaterials is particularly important to manage surface properties. We have investigated the changes in work function resulting from charge transfer between the adsorbent (TM-PICNC) and the adsorbate (CO_2_ molecule). For a semiconductor molecule, the work function refers to the minimum energy required to lift an electron from the Fermi surface to a location that is sufficiently far away from the material’s influence. Meanwhile, the classical equation below theoretically describes the current density of electrons emitted in a vacuum:

$$j={\text{AT}}^{2}\exp\left(\frac{‐\Phi }{\text{kT}}\right)$$ (3)

The constant that relates to the equation describing the current density of electrons emitted in a vacuum is known as Richardson’s constant (A/m^2^), where T represents the temperature in degrees Kelvin, and (eV) is a function of the material. The numerical value of this constant can be calculated using the following equation:

$$\Phi =E_{\inf}–E_{F}$$ (4)

The electrostatic potential at infinity is represented by E_inf_, and the Fermi level energy by E_F_. Assuming E_inf_ to be zero, the calculated values of the function using Eq. 6 for the TM-PICNC molecule when interacting with CO_2_ are provided in the results.

## Results and discussion

### Pristine carbon nanocone (CNC) and porphyrins-induced carbon nanocone (PICNC) geometry optimization

The energy gap value (0.59 eV) between the HOMO and LUMO levels in the carbon nanocone (CNC) structure was found to be low in our calculations. However, our findings indicate that replacing the porphyrin ring in a CNC leads to a slight increase in the energy gap value (1.22 eV) (Table 1). This suggests that the studied structure may tend to act as a semiconductor due to the replacement of the porphyrin ring. Moreover, the binding energy and overall stability of the system do not appear to have significantly changed. Following optimization calculations on the porphyrins that are substituted in the carbon nanocone, PICNC, we have observed that the size of the resulting hole is suitable for accommodating various metals. Furthermore, we anticipate that by binding the nitrogen atoms in this cavity with the atoms of the transition metals, clustering of these metals can be effectively prevented.

**TABLE 1:** Binding energies (E_ads_), energy of HOMO and LUMO orbitals, energy difference of HOMO and LUMO orbitals (E_g_), Fermi level energy (E_F_), and working function (Φ) for CO_2_ molecule, carbon nanocone (CNC), and porphyrins-induced carbon nanocone (PICNC) at the theoretical level of B3LYP/6-31G(d). Values in parentheses refer to single point calculations B3LYP/CC-PVTZ theoretical level.

Systems	E_b_(eV)	E_HOMO_(eV)	E_F_(eV)	E_LUMO_(eV)	E_g_(eV)	Φ
CO_2_	—	−10.07	−4.63	0.81	10.88	4.63
CNC	−6.39	−3.98 (−3.81)	−3.68 (−3.58)	−3.39 (−3.34)	0.59 (0.47)	3.68 (3.58)
PICNC	−6.10	−5.00 (−4.89)	−4.39 (−4.30)	−3.78 (−3.71)	1.22 (1.18)	4.39 (4.30)

Table 1 presents the results obtained from the optimization calculations, which include the energy levels of HOMO and LUMO orbitals, the energy gap (E_g_) between the HOMO and LUMO orbitals, the Fermi level energy, and the work function.

To enhance calculation accuracy, we have incorporated the B3LYP/CC-PVTZ method into the structure of CNC and PICNC through single point calculations. The corresponding findings are presented in Table 1, highlighting the remarkable alignment of these results with our own. This methodology bolsters our outcomes, lending further credibility to our findings.

### Structural study of metalloporphyrins-induced in carbon nanocone (TM-PICNC)

**FIGURE 1:**
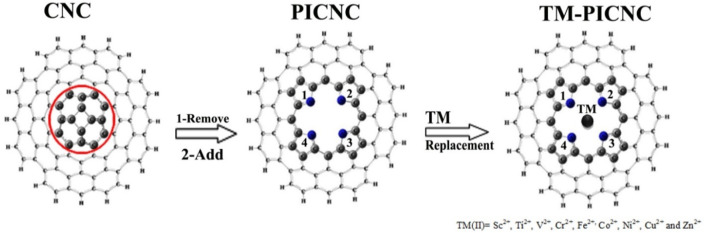
A schematic formation of porphyrins and metallic porphyrins-induced in carbon nanocones.

Depending on the size of the porphyrin central cavity and the type of metal ions, first-order transition metal cations (TM(II)) can be positioned at the center of the cavity. These metal ions may include scandium (Sc^2+^), titanium (Ti^2+^), vanadium (V^2+^), chromium (Cr^2+^), iron (Fe^2+^), cobalt (Co^2+^), nickel (Ni^2+^), copper (Cu^2+^), and zinc (Zn^2+^). To investigate the effects of porphyrin-induced defects on carbon nanocones, first-order divalent metal ions of the transition elements were replaced at the center of the porphyrin molecule in carbon nanocones. Subsequently, optimization calculations were performed for the nanocone (CNC), the porphyrin ring-induced model (PICNC), and the metallic porphyrin-induced model in carbon nanocones (TM-PICNC), as depicted in Figure 1.

Table 1 demonstrates that the HOMO and LUMO orbitals in porphyrin-induced carbon nanocones, TM-PICNC, undergo changes after the first-order divalent transition metal ions are placed in the empty cavity of the molecule. It is important to note that replacing Sc and Ti metal ions in the porphyrin central cavity results in a significant decrease in, E_g_ compared to isolate PICNC. However, this change is increased for metal ions of Cr, Fe, Co, Ni, Cu, and Zn in PICNC. These findings suggest that the placement of TM cations in PICNC causes a shift in the energy levels between the HOMO and LUMO orbitals. Certainly, these results suggest that the introduction of the mentioned cations into PICNC leads to the formation of a new HOMO and LUMO orbitals with higher energy levels. Specifically, Sc-PICNC, Ti-PICNC, and V-PICNC have, E_g_ values of 0.70, 0.83, and 1.14 eV, respectively. Meanwhile, Cr-PICNC, Fe-PICNC, Co-PICNC, Ni-PICNC, Cu-PICNC, and Zn-PICNC have, E_g_ values of 1.43, 2.00, 1.96, 1.98, 1.95, and 1.96 eV, respectively. Furthermore, we conducted single point calculations at the B3LYP/CC-PVTZ level for the V-PICNC configuration, bolstering the validity of our findings (See Table 2). The interaction between a porphyrin induced with vanadium was thoroughly examined in our study, employing an exceptionally large nanocone (a nanocone C_300_H_37_N_4_ with a cone angle of 83.6° and a length of 13.96 Å). Detailed findings from this investigation can be found in Table 2.

**TABLE 2:** Energy of HOMO and LUMO orbitals, energy difference of HOMO and LUMO orbitals (E_g_), Fermi level energy (E_F_), and working function (Φ) for (TM-PICNC) at the theoretical level of B3LYP/6-31G(d).Values in parentheses refer to single point calculations B3LYP/CC-PVTZ theoretical level.

Systems	E_HOMO_ (eV)	E_F_ (eV)	E_LUMO_ (eV)	E_g_ (eV)	Φ (eV)
Sc-PICNC	−3.71	−3.36	−3.01	0.70	3.36
Ti-PICNC	−3.92	−3.51	−3.09	0.83	3.51
V-PICNC	−4.20 (−4.05)	−3.63 (−3.49)	−3.06 (−2.93)	1.14 (1.12)	3.63 (3.49)
V-LPICNC^a^	−3.80 (−3.71)	−3.19 (−3.07)	−2.57 (−2.43)	1.23 (1.28)	3.19 (3.07)
Cr-PICNC	−4.43	−3.72	−3.00	1.43	3.72
Fe-PICNC	−4.84	−3.84	−2.84	2.00	3.84
Co-PICNC	−4.86	−3.89	−2.91	1.96	3.89
Ni-PICNC	−4.86	−3.88	−2.89	1.98	3.88
Cu-PICNC	−4.85	−3.88	−2.90	1.95	3.88
Zn-PICNC	−4.85	−3.87	−2.89	1.96	3.87

^a^ Larger porphyrin induced nanocone C_300_H_37_N_4_ with a cone angle of 83.6° and a length of 13.96 Å.

As a result of the absorption of metal ions, the Fermi level changes, and this change is higher for some ions compared to others, such as (Fe, Co, Ni, Cu) ions. The energy gap value of porphyrin molecule in carbon nanocone is around 1.22 (eV), which makes the studied compound tend to be a semiconductor. The change in the Fermi level of a semiconductor during the absorption of metal ions alters the field emission currents.

**FIGURE 2:**
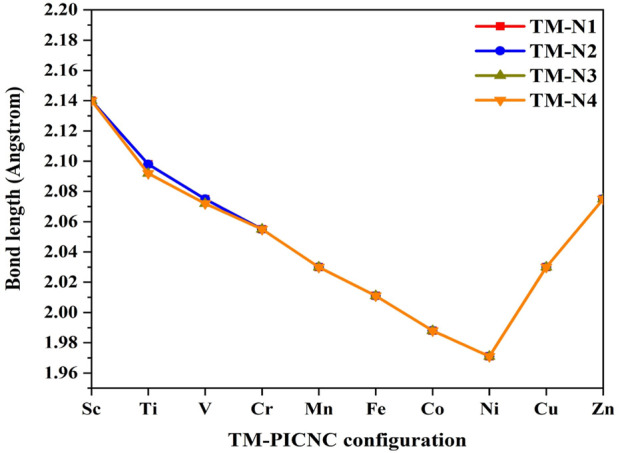
Bond length obtained from optimization calculations at the theoretical level B3LYP/6-31G(d) related to TM-PICNC.

As mentioned earlier, the hole created in the center of the porphyrin ring is such that a variety of first-order metals can be used as transition elements. The results of Figure 2 show that by placing the metal ions TM = Sc, Ti, V, Cr, Fe, Co, Ni, Cu, Zn in the center of the porphyrin cavity, the metal ions are located exactly in the center of the porphyrin cavity. DFT calculations show that the TM-N bond length for TM = Ti, V, Cr, Fe, Co, Ni, Cu, Zn is shorter than Sc-N, indicating a stronger interaction with porphyrin replaced in nanocones.

### Adsorption of CO_2_ molecule on TM-PICNC

**FIGURE 3:**
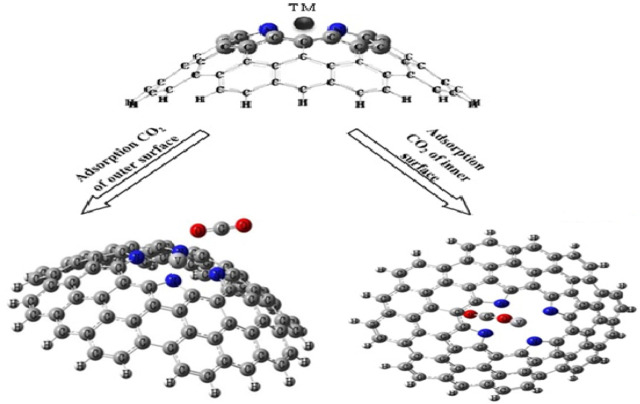
Schematic of initial CO_2_ molecule adsorption orientation on transition metal ions porphyrin-induced carbon nanocone surfaces (TM-PICNC).

We have examined the interaction between one CO_2_ gas and one TM-PICNC (1:1). In order to determine the optimal adsorption energy, we have analyzed the interaction of CO_2_ from both internal and external sites with the metal ions present in TM-PICNC (as shown in Figure 3).

**FIGURE 4:**
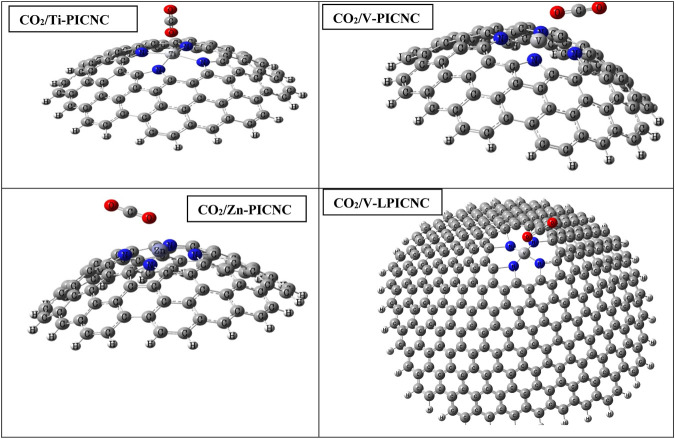
Optimized structures for the adsorption of CO_2_ molecules from the outer surface of TM-PICNC.

**FIGURE 5:**
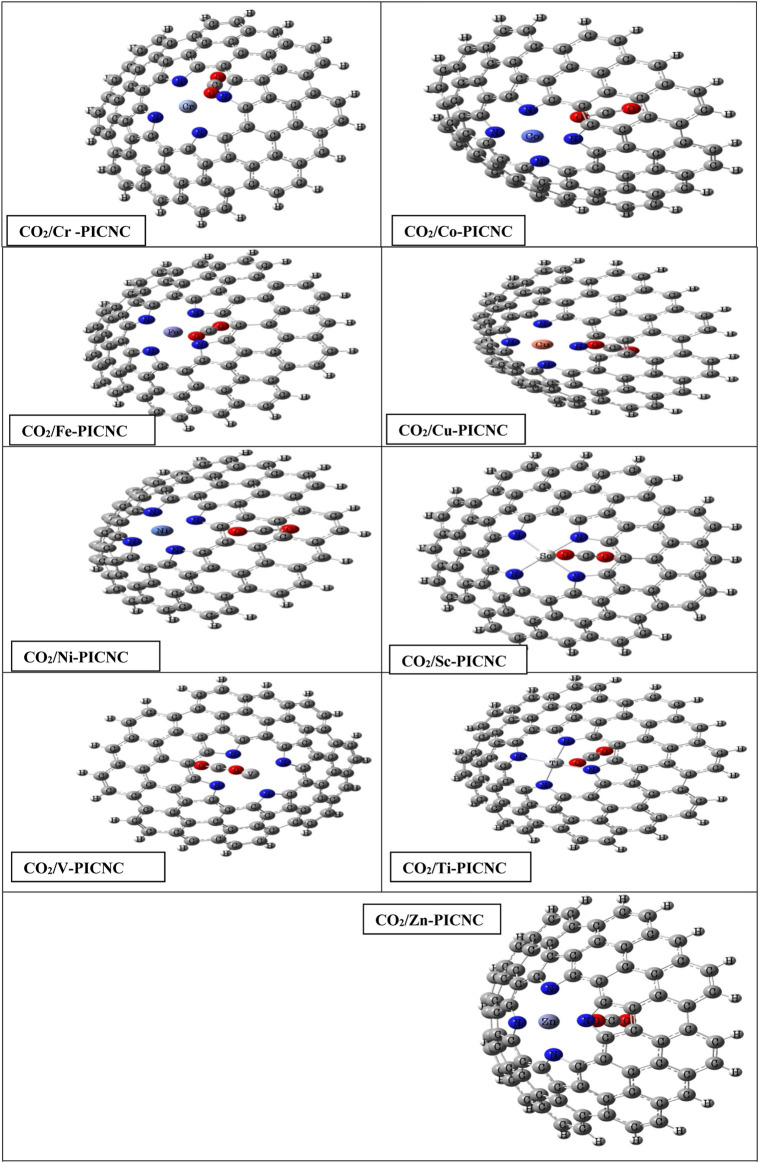
Optimized structures for the adsorption of the CO_2_ molecules from the inner surface of TM-PICNC.

The interactions between CO_2_ gas and TM-PICNC are depicted in Figure 3, illustrating the gas positioned in the center of the porphyrin ring from both internal and external sites. The electrostatic properties of the CO_2_ gas and the outer and inner surfaces of TM-PICNC (where TM = Sc^2+^, Ti^2+^, V^2+^, Cr^2+^, Fe^2+^, Co^2+^, Ni^2+^, Cu^2+^, Zn^2+^) were studied through DFT calculations using the B3LYP method and 6-31G(d) basis set. To determine the most stable adsorption configuration, various dihedral angles were scanned (Figures 4, 5). Table 3 displays the E_ads_ values computed for the adsorption of CO_2_ onto TM-PICNC molecules using Eq. 1.

**TABLE 3:** Absorption energies (E_ads_), energy of HOMO and LUMO orbitals, energy difference of HOMO and LUMO orbitals (E_g_), Fermi level energy (E_F_), and working function for (TM-PICNC) after adsorption of CO_2_ molecule, O_2_ and H_2_O adsorption over the V-PICNC at the theoretical level. Values in parentheses refer to single point calculations B3LYP/CC-PVTZ theoretical level.

Systems	Orientation	E_ads_ (kCal/mol)	E_HOMO_ (eV)	E_F_ (eV)	E_LUMO_ (eV)	E_g_ (eV)	%ΔE_g_	Ф (eV)	%ΔФ
Sc-PICNC	CO_2_-out	−12.12	−3.58	−3.23	−2.89	0.69	1.51	3.23	3.75
Sc-PICNC	CO_2_-in	−6.59	−3.73	−3.38	−3.03	0.70	0.31	3.38	0.64
Ti-PICNC	CO_2_-out	−11.80	−3.95	−3.49	−3.02	0.94	12.85	3.49	0.52
Ti-PICNC	CO_2_-in	−5.06	−4.06	−3.59	−3.12	0.94	13.02	3.59	2.37
V-PICNC	CO_2_-out	−9.13 (−8.90)	−4.82 (−4.70)	−3.93 (−3.82)	−3.04 (−2.95)	1.78 (1.75)	56.79 (56.25)	3.93 (3.82)	8.29 (9.46)
V-PICNC	CO_2_-in	−0.33	−4.92	−4.28	−3.64	1.28	10.14	4.28	17.83
V-PICNC	O_2_-out	−2.79	−4.98	−4.39	−3.80	1.18	3.51	4.39	20.94
V-PICNC	H-H_2_O-out	−1.94	−5.06	−4.49	−3.91	1.15	0.88	4.49	23.69
V-PICNC	O-H_2_O-out	−3.19	−5.10	−4.52	−3.93	1.17	2.63	4.52	23.97
V-LPICNC^a^	CO_2_-out	−8.60 (−8.29)	−4.39(-4.27)	−3.65(-3.52)	−2.90 (−2.76)	1.49 (1.51)	21.14(17.97)	3.65(3.52)	14.42(14.66)
Cr-PICNC	CO_2_-out	0.80	−4.56	−3.73	−2.90	1.66	15.59	3.73	0.41
Cr-PICNC	CO_2_-in	0.66	−4.42	−3.71	−3.00	1.41	1.37	3.71	0.10
Fe-PICNC	CO_2_-out	−6.58	−4.79	−3.79	−2.79	2.00	1.05	3.79	1.37
Fe-PICNC	CO_2_-in	−4.73	−4.84	−3.84	−2.84	2.00	0.08	3.84	0.02
Co-PICNC	CO_2_-out	−4.13	−4.83	−3.84	−2.85	1.98	1.35	3.84	1.15
Co-PICNC	CO_2_-in	1.05	−4.86	−3.87	−2.89	1.98	1.03	3.87	0.30
Ni-PICNC	CO_2_-out	0.03	−4.87	−3.88	−2.89	1.98	0.03	3.88	0.15
Ni-PICNC	CO_2_-in	0.47	−4.86	−3.87	−2.89	1.97	0.91	3.87	0.02
Cu-PICNC	CO_2_-out	0.03	−4.85	−3.87	−2.90	1.95	0.03	3.87	0.04
Cu-PICNC	CO_2_-in	0.24	−4.85	−3.88	−2.90	1.95	0.28	3.88	0.03
Zn-PICNC	CO_2_-out	−1.55	−4.80	−3.83	−2.85	1.95	0.19	3.83	1.09
Zn-PICNC	CO_2_-in	0.13	−4.84	−3.87	−2.89	1.95	0.32	3.87	0.06

^a^ Larger porphyrin induced nanocone C_300_H_37_N_4_ with a cone angle of 83.6° and a length of 13.96 Å.

Based on Table 3, the configurations can be classified into three groups, with the first group consisting of Sc-PICNC, Ti-PICNC, and V-PICNC. These structures interact with CO_2_ gas on their outer surface and are found to be stable. The energy of adsorption (E_ads_) for these configurations was computed to be −12.12, −11.80, and −9.13 kcal/mol, respectively, which confirms the strong physical adsorption of CO_2_ gas on the exterior surface of TM-PICNC (where TM = Sc^2+^, Ti^2+^, and V^2+^). This suggests that CO_2_ gas molecules can interact with the O of the gas molecule and the metal atom from TM-PICNC structures, resulting in the configuration’s stability (as depicted in Figure 4). The O-TM interaction distances for these configurations fall within the range of 2.17–2.29 Å (refer to Table 4).

**TABLE 4:** Calculated O=C=O angle in B3LYP/6-31G* level of theory.

Sc-PICNC	CO_2_-out	175.2
Sc-PICNC	CO_2_-in	178.6
Ti-PICNC	CO_2_-out	176.5
Ti-PICNC	CO_2_-in	179.1
V-PICNC	CO_2_-out	178.2
V-PICNC	CO_2_-in	179.7
V-LPICNC*	CO_2_-out	178.6
Cr-PICNC	CO_2_-out	179.8
Cr-PICNC	CO_2_-in	179.9
Fe-PICNC	CO_2_-out	178.9
Fe-PICNC	CO_2_-in	179.4
Co-PICNC	CO_2_-out	179.1
Co-PICNC	CO_2_-in	180
Ni-PICNC	CO_2_-out	180
Ni-PICNC	CO_2_-in	179.9
Cu-PICNC	CO_2_-out	179.9
Cu-PICNC	CO_2_-in	179.8
Zn-PICNC	CO_2_-out	179.4
Zn-PICNC	CO_2_-in	179.8

The larger porphyrin induced nanocone (V-LPICNC) interact with CO_2_ gas on its outer surface with E_ads_ of −8.60 kCal/mol. This nanocone is a weak sensor for CO_2_ gas with its physical adsorption and relatively low %ΔE_g_.

The second group of configurations exhibit weak adsorption energy, including the Sc-PICNC, Ti-PICNC, V-PICNC, and Fe-PICNC configurations with CO_2_ gas adsorbed on their inner surface, as well as CO_2_ gas interaction on the outer surface of Fe-PICNC, Co-PICNC, and Zn-PICNC in Table 3. The E_ads_ values for these configurations, ranging from −6.59 to −0.33 kcal/mol, indicate a weak physical adsorption of CO_2_ gas on the TM-PICNC (where TM = Sc^2+^, Ti^2+^, V^2+^, Fe^2+^, Co^2+^, and Zn^2+^) surface. The interaction distance between O from CO_2_ gas and TM TM-PICNC ranges from 2.03–2.51 Å, confirming their weak interaction. The last category of structures results from the interaction between CO_2_ gas and the absorbent shown in Figures 4, 5. Here, the O atom of CO_2_ gas interacts with the TM atom of TM-PICNC (TM = Cr^2+^, Co^2+^, Ni^2+^, Cu^2+^, and Zn^2+^) with O ^…^ TM distances ranging from about 2.27 to 4.37 Å (Table 5). The calculated Eads values for these configurations, ranging from 0.03 to 1.05 kcal/mol (Table 3), indicate that the interaction between CO_2_ gas and these adsorbents is a very weak physisorption process, as positive E_ads_ values suggest instability. Table 4 also reports the change in the angles of CO_2_ gas, and from the results of the table, we can see that the O=C=O angle changes are between 175.2 and 180°.

**TABLE 5:** Bond length and angle bonds obtained from optimization calculations at the theoretical level B3LYP/6-31G(d) related to TM-PICNC after CO_2_ adsorption process.

Systems	Orientation	TM-N1	TM-N2	TM-N3	TM-N4	TM-O5	O5-C6	C6-O7	TM-O5-C6	O5-C6-O7
Sc-PICNC										
	CO_2_-out	2.153	2.153	2.153	2.153	2.288	1.176	1.157	177.14	179.85
	CO_2_-in	2.127	2.128	2.127	2.131	2.379	1.176	1.159	149.32	178.52
Ti-PICNC										
	CO_2_-out	2.070	2.102	2.070	2.102	2.166	1.175	1.161	179.90	179.99
	CO_2_-in	2.069	2.072	2.069	2.076	2.263	1.175	1.160	151.05	177.84
V-PICNC										
	CO_2_-out	2.136	2.059	2.078	2.059	2.216	1.182	1.158	126.40	176.58
	CO_2_-in	2.061	2.063	2.065	2.064	2.187	1.176	1.159	147.23	177.90
Cr-PICNC										
	CO_2_-out	2.026	2.060	2.139	2.061	2.266	1.179	1.161	125.20	176.82
	CO_2_-in	2.055	2.056	2.055	2.056	4.370	1.169	1.169	162.69	179.63
Fe-PICNC										
	CO_2_-out	2.014	2.018	2.029	2.018	2.025	1.177	1.160	131.53	176.51
	CO_2_-in	2.015	2.015	2.015	2.015	2.047	1.174	1.162	146.14	177.35
Co-PICNC										
	CO_2_-out	2.004	1.996	1.996	2.005	2.512	1.173	1.165	112.84	179.01
	CO_2_-in	2.002	1.989	2.002	1.989	3.629	1.169	1.169	143.55	179.74
Ni-PICNC										
	CO_2_-out	1.973	1.970	1.970	1.973	3.360	1.170	1.169	19.14	179.06
	CO_2_-in	1.971	1.971	1.970	1.971	3.636	1.169	1.169	168.33	179.81
Cu-PICNC										
	CO_2_-out	2.042	2.032	2.032	2.033	2.886	1.171	1.167	108.61	179.08
	CO_2_-in	2.029	2.030	2.029	2.030	3.353	1.169	1.168	166.65	179.80
Zn-PICNC										
	CO_2_-out	2.080	2.087	2.116	2.087	2.403	1.175	1.163	18.31	178.44
	CO_2_-in	2.075	2.075	2.076	2.075	3.147	1.169	1.168	173.09	179.89

Our investigation also focused on electronic properties, particularly E_HOMO_, E_LUMO_, and, E_g_, which are important parameters that can explain the behavior of the molecules. Table 3 shows that the change in, E_g_ for the configurations obtained from CO_2_ adsorption on the outer and inner surface of Ti-PICNC, V-PICNC, and from CO_2_ adsorption on the outer surface of Cr-PICNC, compared to the respective adsorbents, is significant (%∆E = 12.85, 13.02, 56.79, 10.14, and 15.59), indicating strong adsorption. The significant change in, E_g_ is due to the new HOMO and LUMO levels of these configurations compared to their adsorbents. For other configurations in Table 3, the shift in, E_g_ (%∆E = ranges between 0.03-1.51) is negligible, indicating weak adsorption on the exterior and interior surface of these adsorbents.

**FIGURE 6:**
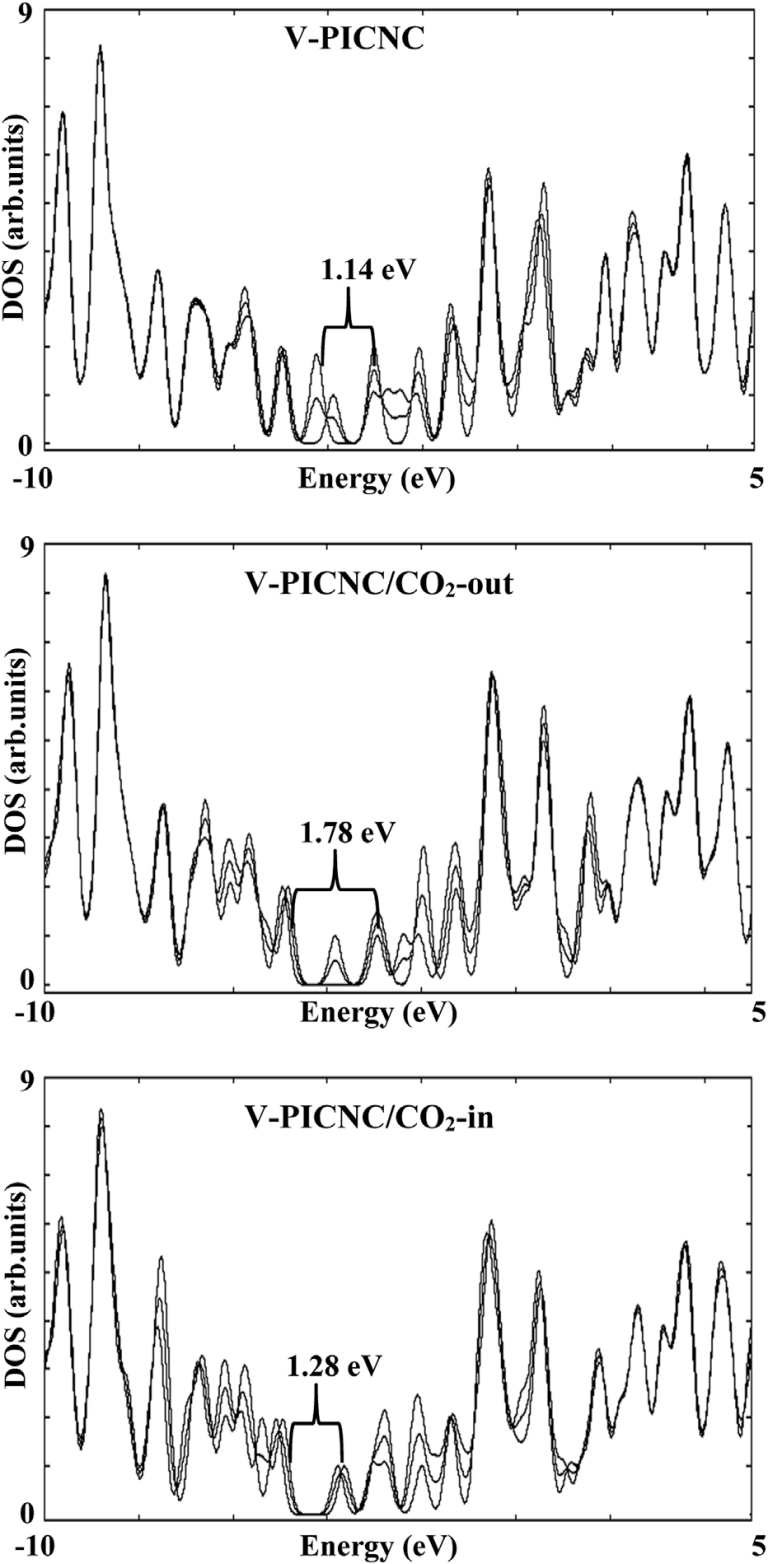
The density of state (DOS) for V-PICNC before and after the adsorption of CO_2_ molecules from the internal and external surfaces.

We also analyzed the electronic properties of V-PICNC and its CO_2_ adsorption configurations on the outer and inner surface, as presented in Table 3 and Figure 6. Our results show a remarkable change in the DOS of the considered configurations compared to V-PICNC, which is related to the physisorption process. Specifically, we observe new LUMO and HOMO levels due to the interaction between CO_2_ gas and V-PICNC. Furthermore, the DOS plots indicate that the valence and conduction levels in both the V-PICNC/CO_2_-out and V-PICNC/CO_2_-in configurations shift notably downwards, leading to a significant increase in the, E_g_ value of V-PICNC by 56.79% and 10.14%, respectively (especially in V-PICNC/CO_2_-out).

According to the obtained results, the Oxygen gas molecule has been approached from the external surface of V-PICNC. The absorption was weak and physical (−2.79 kcal/mol), which had little changes on %ΔE_g_ about 3.51%. This work was repeated for H_2_O from both O and H terminations, and again physical absorption (E_ads_ = −3.19 and −1.94 kcal/mol, respectively) and slight changes in %ΔE_g_ (2.63% and 0.88%, respectively) were observed. The general result is that V-PICNC is not only unable to sense Oxygen and water, but also desorbs them quickly. Therefore, the presence of Oxygen gas and H_2_O molecule does not create a problem for the sense of CO_2_ gas by TM-PICNC.

As depicted in Table 4; Figure 4, the findings obtained through the utilization of the single point calculations B3LYP/CC-PVTZ method have been documented for the V-PICNC configuration, thereby corroborating the outcomes derived from our methodology.

In terms of gas sensing potential, two important parameters are Eads and HOMO-LUMO energy gap (E_g_). The adsorption of CO_2_ gas over TM-PICNC can be reversible if the E_ads_ falls within a suitable range. Stronger interactions, however, are not favorable for CO_2_ gas sensing due to a high recovery time and the consequent difficulty in desorbing CO_2_ gas over TM-PICNC.

As E_ads_ becomes more negative, the CO_2_ adsorption over TM-PICNC becomes stronger, which can result in a longer recovery time (τ). This relationship can be described using the following equation(Redondo et al., 1983; Kumar et al., 2017):

$$\tau =\nu_{0}^{–1}\exp\left(–E_{\text{ads}}/\text{kT}\right)$$ (5)

The equation provided relates recovery time (τ) and attempt frequency (ν_0_) to temperature (T) and Boltzmann constant (k) (Bano et al., 2019). Equation 5 shows an exponential correlation between E_ads_ and recovery time. Sensor recovery is a critical process that can operate at room temperature or higher, according to some sources (Li et al., 2003). The second crucial parameter that affects the sensing ability of TM-PICNC is the, E_g_ in the presence of CO_2_ gas. As presented in Eq. 6, E_g_ is directly proportional to the conduction electron population (σ), which increases when CO_2_ gas is adsorbed onto TM-PICNC, resulting in a decrease in the HOMO-LUMO energy gap (E_g_). Conversely, increasing the value of %∆E_g_ also increases the sensing potential. The correlation between, E_g_ and electrical conductance of nanoparticles can be expressed as follows:

$$\sigma =AT^{3/2}\exp\left(–E_{g}/2\text{kT}\right)$$ (6)

This equation involves the constant A (electrons/m^3^K^3/2^) and the Boltzmann constant k. Furthermore, the results obtained from this procedure show a noticeable correlation with the experimental techniques described in scientific literature (Hadipour et al., 2015). The sensitivity of TM-PICNC to CO_2_ gas is established by the equation given in Eq. 6. Hence, the electrical conductivity can be converted into an electrical signal upon the presence of gas molecules. (Beheshtian et al., 2012). The data presented above suggests that Ti-PICNC/CO_2_-out and V-PICNC/CO_2_-out may be promising candidates for physisorption-based CO_2_ gas sensing.

The calculated values of the work function using Eq. 4 for the TM-PICNC molecule when interacting with CO_2_ are provided in Table 3. The DFT calculations revealed that the calculated values of TM-PICNC molecules changed following CO_2_ adsorption. Equation 3 indicates that the electron diffusion current density is exponentially dependent on a negative value. Since the Fermi level of TM-PICNC is not significantly altered, the current density slowly changes upon CO_2_ adsorption, indicating low sensitivity of the adsorbent to the presence of CO_2_. The %∆Ф values of V-PICNC at the inner and outer sites are 17.83% and 8.29%, respectively, indicating higher sensing ability. Nonetheless, V-PICNC may function as an Φ-type sensor due to its physical adsorption energy and low recovery time.

### NBO analysis

Table 6 reports the results of the NBO analysis, which involved evaluating all possible interactions between filled Lewis-type NBOs (donors) and empty non-Lewis NBOs (acceptors). These interactions, known as delocalization corrections, were included to refine the zeroth-order natural Lewis structure. The table lists the stabilization energies (E^(2)^) for the most important interaction between electron-donor orbitals (i) and electron-acceptor orbitals (j). A large value of E^(2)^ indicates strong interaction between the V metal ion and the PICNC molecule, suggesting that they are more likely to transfer electrons from the donor orbital (i) to the acceptor orbital (j).

**TABLE 6:** Part of the second-order perturbation of the stabilizing energy calculated for the donor-acceptor natural orbitals for V-PICNC.

System	Orientation	Donor NBO (*i*)	Acceptor NBO (*j*)	E ^(2)^ kcal/mol
V-PICNC	-			
		LP N(1)	LP*V	59.40
		LP N(2)	LP*V	59.34
		LP N(3)	LP*V	59.30
		LP N(4)	LP*V	59.44
V-PICNC	CO_2_-out	LP N(1)	LP*V	51.07
		LP N(2)	LP*V	54.94
		LP N(3)	LP*V	65.3
		LP N(4)	LP*V	65.2
		LP O(5)	LP*V	36.06
V-PICNC	CO_2_-in	LP N(1)	LP*V	63.91
		LP N(2)	LP*V	62.22
		LP N(3)	LP*V	63.45
		LP N(4)	LP*V	62.57
		LP O(5)	LP*V	39.26

To further investigate the matter, we analyzed the degree of stabilization energy (E^(2)^), as well as the electron donor orbitals (i) and electron acceptor orbitals (j) when the CO_2_ molecule is in proximity to the V metal ion. Our results indicate that the primary electron donor orbitals (i) and electron acceptor orbitals (j) that result in high stabilization energy are LP_N(3)_ => LP*_V_ and LP_N(4)_ => LP*_V_, LPN_(1)_ => LP*_V_ and LP_N(3)_ => LP*_V_ for the CO_2_-out and CO_2_-in orientations, respectively. In the LP_N_(n) => LP*_V_ interaction, the lone pair electrons of the N atom transfer to the lone pair anti-bonding orbital of the V metal ion, which is consistent with the results obtained from FMO analysis.

The Wiberg bond index (WBI) is a measure that characterizes the strength and type of chemical bonds. It is derived from the density matrix obtained through the NBO (Natural Bond Orbital) analysis using orthogonal natural orbitals. Mathematically, the WBI is calculated as the sum of the squared density matrix elements (p_jk_), which is equivalent to twice the charge density in atomic orbitals (P_jj_) minus the square of the charge density, expressed by the following formula:

$$WBI=\sum_{k}p_{jk}^{2}=2p_{jj}-p_{jj}^{2}$$ (7)

**FIGURE 7:**
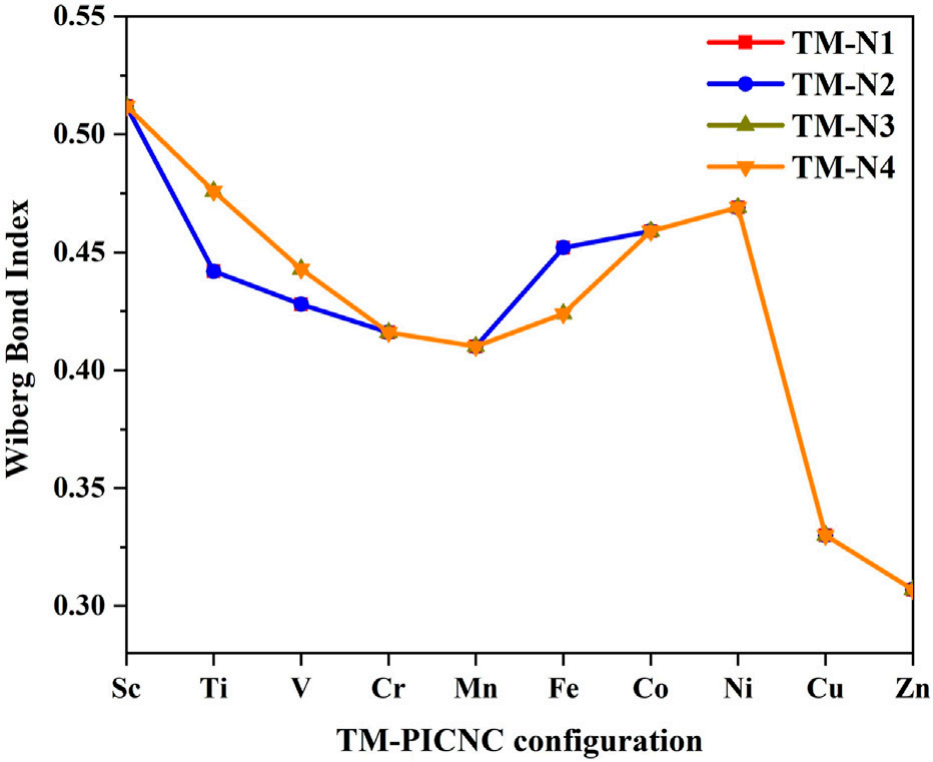
Wiberg bond index for metal-nitrogen bonds (TM-N) in metal porphyrins replaced in carbon nano cones.

Figure 7 lists the findings of the NBO analysis, which was conducted to examine and determine the nature of the metal bonds in TM-PICNC, using the Wiberg bond index. According to the analysis, dative bonds are typically characterized by a Wiberg index ranging from 0.3 to 0.6, which suggests that the bonds between TM and N in this case are dative in nature. The results indicate that the Wiberg index for the 4 N ^…^ SC dative bonds is 0.512 (Figure 7). The Wiberg bond index for each of the four V-N bonds in the studied compound is equal to 0.428-0.443. This value decreases to 0.307 when moving across the periodic table and replacing V with Cu or Zn, as well as when substituting these metals into the central cavity of the first-row transition metal ring. These data and results indicate the presence of a dative bond between nitrogen atoms and metal ions.

In continuation, the Wiberg bond index for the TM-N bond in the TM-PICNC compound was investigated. The index was examined in Table 7 for the case where the central metal of the porphyrin cavity interacts with CO_2_ molecule in a carbon nanocone. The investigations carried out in Table 7 indicate that when a CO_2_ gas molecule is adsorbed onto the first-row transition metal ions porphyrins that have been induced in a carbon nanocone, the metal-nitrogen (TM-N) bonds of the first-row transition metal porphyrins that have been induced in a carbon nanocone remain dative in nature and are in the range of 0.3–0.6. However, the value of the Wiberg bond index for the bond between the first-row transition metal and the oxygen atom in the CO_2_ molecule (TM-O) shows that the adsorption of CO_2_ gas molecule on the substituted first-row transition metal porphyrins in the carbon nanocone is variable, and the adsorption of CO_2_ gas molecule on the induced porphyrins in the carbon nanocone is within a physical range.

**TABLE 7:** The Wiberg bond index for the metal-nitrogen bonds and the metal-oxygen bonds of TM-PICNC during CO2 adsorption process.

Systems	Orientation	TM-N1	TM-N2	TM-N3	TM-N4	TM-O5	O5-C6	C6-O7
Sc-PICNC								
	CO_2_-out	0.447	0.448	0.447	0.448	0.198	1.717	1.9982
	CO_2_-in	0.428	0.427	0.425	0.424	0.167	1.751	1.9768
Ti-PICNC								
	CO_2_-out	0.548	0.558	0.558	0.548	0.248	1.705	1.9705
	CO_2_-in	0.520	0.553	0.520	0.557	0.198	1.740	1.9728
V-PICNC								
	CO_2_-out	0.402	0.531	0.468	0.531	0.205	1.715	1.9953
	CO_2_-in	0.485	0.483	0.482	0.481	0.214	1.727	1.986
Cr-PICNC								
	CO_2_-out	0.479	0.444	0.380	0.444	0.176	1.748	1.966
	CO_2_-in	0.429	0.429	0.429	0.428	0.004	1.885	1.8897
Fe-PICNC								
	CO_2_-out	0.432	0.429	0.423	0.429	0.237	1.733	1.9716
	CO_2_-in	0.421	0.421	0.422	0.421	0.222	1.742	1.9626
Co-PICNC								
	CO_2_-out	0.435	0.440	0.440	0.434	0.090	1.824	1.9242
	CO_2_-in	0.430	0.437	0.430	0.437	0.016	1.883	1.8909
Ni-PICNC								
	CO_2_-out	0.458	0.461	0.461	0.458	0.020	1.880	1.8889
	CO_2_-in	0.462	0.463	0.462	0.461	0.014	1.885	1.8894
Cu-PICNC								
	CO_2_-out	0.314	0.317	0.318	0.317	0.050	1.856	1.9051
	CO_2_-in	0.319	0.320	0.319	0.318	0.025	1.877	1.8946
Zn-PICNC								
	CO_2_-out	0.297	0.295	0.285	0.295	0.111	1.795	1.9439
	CO_2_-in	0.294	0.294	0.294	0.294	0.036	1.867	1.9001

## Conclusion

Density functional theory (DFT) was used to investigate the adsorption of a CO_2_ molecule on transition metal ions (TM) porphyrins induced carbon nanocone (TM-PICNC), where TM = Sc^2+^, Ti^2+^, V^2+^, Cr^2+^, Fe^2+^, Co^2+^, Ni^2+^, Cu^2+^, and Zn^2+^. The results showed that the CO_2_ molecule is adsorbed on TM-PICNC with adsorption energies ranging from 0.03 to −12.12 kcal/mol. The strongest interaction of CO_2_ gas was observed over Sc, Ti, and V-PICNC from the outer site with E_ads_ = −12.12, −11.80, and −9.13 kcal/mol, respectively, whereas the weakest interaction was found for Cu and Ni-PICNC from both outer and inner sites. The, E_g_ of Ti-PICNC/CO_2_-out and -in, V-PICNC/CO_2_-out and -in, and Cr-PICNC/CO_2_-out showed significant changes compared to others. In determining the sensing ability, two parameters, E_ads_ and, E_g_, are important. The physical and reversible absorption of CO_2_ gas over TM-PICNC leading to a medium amount of E_ads_ is suitable, while a high change in, E_g_ is beneficial for promising sensing ability. Based on these criteria, V-PICNC with E_ads_ = -9.13 kcal/mol and %ΔE_g_ 56.79 is a good candidate for detecting the presence of CO2 molecule. Additionally, the ΔФ value for V-PICNC from both outer and inner sites is 8.29 and 17.83, respectively, indicating higher sensing ability. It is suggested that V-PICNC can serve as an Φ-type sensor due to its physical absorption energy and low recovery time. In addition, computaional evidence demonstrated that the TM-PICNC lacks the necessary sensitivity to detect water and oxygen molecules.

## Data Availability

The original contributions presented in the study are included in the article/Supplementary Material, further inquiries can be directed to the corresponding author.
